# Down-regulation of cellular FLICE-inhibitory protein (Long Form) contributes to apoptosis induced by Hsp90 inhibition in human lung cancer cells

**DOI:** 10.1186/1475-2867-12-54

**Published:** 2012-12-21

**Authors:** Qilin Wang, Wendong Sun, Xuexi Hao, Tianliang Li, Ling Su, Xiangguo Liu

**Affiliations:** 1 Key Laboratory for Experimental Teratology of the Ministry of Education and School of Life Sciences, Shandong University, Jinan, China; 2 Liaocheng University School of Life Sciences, Liaocheng, China; 3 The Second Hospital, Shandong University, Jinan, China; 4 Shandong University School of Life Sciences, Room103, South Building, 27 Shandananlu Road, Jinan, 250100, China

**Keywords:** c-FLIP_L_, Apoptosis, CHIP, Hsp90

## Abstract

**Background:**

Cellular FLICE-Inhibitory Protein (long form, c-FLIP_L_) is a critical negative regulator of death receptor-mediated apoptosis. Overexpression of c-FLIP_L_ has been reported in many cancer cell lines and is associated with chemoresistance. In contrast, down-regulation of c-FLIP may drive cancer cells into cellular apoptosis. This study aims to demonstrate that inhibition of the heat shock protein 90 (Hsp90) either by inhibitors geldanamycin/17-N-Allylamino-17-demethoxygeldanamycin (GA/17-AAG) or siRNA technique in human lung cancer cells induces c-FLIP_L_ degradation and cellular apoptosis through C-terminus of Hsp70-interacting protein (CHIP)-mediated mechanisms.

**Methods:**

Calu-1 and H157 cell lines (including H157-c-FLIP_L_ overexpressing c-FLIP_L_ and control cell H157-lacZ) were treated with 17-AAG and the cell lysates were prepared to detect the given proteins by Western Blot and the cell survival was assayed by SRB assay. CHIP and Hsp90 α/β proteins were knocked down by siRNA technique. CHIP and c-FLIP_L_ plasmids were transfected into cells and immunoprecipitation experiments were performed to testify the interactions between c-FLIP_L_, CHIP and Hsp90.

**Results:**

c-FLIP_L_ down-regulation induced by 17-AAG can be reversed with the proteasome inhibitor MG132, which suggested that c-FLIP_L_ degradation is mediated by a ubiquitin-proteasome system. Inhibition of Hsp90α/β reduced c-FLIP_L_ level, whereas knocking down CHIP expression with siRNA technique inhibited c-FLIP_L_ degradation. Furthermore, c-FLIP_L_ and CHIP were co-precipitated in the IP complexes. In addition, overexpression of c-FLIP_L_ can rescue cancer cells from apoptosis. When 17-AAG was combined with an anti-cancer agent celecoxib(CCB), c-FLIP_L_ level declined further and there was a higher degree of caspase activation.

**Conclusion:**

We have elucidated c-FLIP_L_ degradation contributes to apoptosis induced by Hsp90 inhibition, suggesting c-FLIP and Hsp90 may be the promising combined targets in human lung cancer treatment.

## Introduction

Caspase-8 activation plays an important role in the death receptor-mediated extrinsic apoptotic pathway in human cancer cells [1]. When binding to ligands, the death receptor is activated and forms the death induced signal complex (DISC) together with FADD and procaspase-8. Procaspase-8 is activated and initiates the caspase cascade which mediates cellular apoptosis. c-FLIP_L_ is a major protein that can prevent caspase-8/10 activation in the DISC and inhibiting apoptosis mediated by death receptors. It has been found that c-FLIP_L_ is up-regulated in several carcinomas [2,3] and overexpression of c-FLIP_L_ can be responsible for chemoresistance and malignant transformation [4-6]. To date, more than 10 splicing variants of c-FLIP genes have been identified at the mRNA level. However, only c-FLIP_L_ and c-FLIP_S_ have been extensively studied at the protein level. Although these two splicing variants have distinct structural and functional properties, they have been found to be recruited to DISC and to inhibit caspase-8 activation. Recent studies have shown that c-FLIP_L_ degradation is dependent on JNK-mediated E3-ligase Itch and AKT phosphorylation, while degradation of c-FLIP_S_ seems to have different pattern [7,8].

Hsp90 is a pleiotropic molecular chaperone and functions as a key protein in the conformational maturation and stability of client proteins, many of which are kinases, cell cycle regulators and steroid receptors, etc. [9]. These client proteins play important roles in signaling transduction, cell proliferation and cancer chemoresistance. The Hsp90-based molecular chaperone complex interacts with its client proteins in an iterative way. Hsp90 cycles are driven by ATP- or ADP-bound conformations that are mediated via multiple rounds of ATP binding and hydrolysis [9]. Hsp90 inhibitors such as geldanamycin (GA) or its synthetic analogue 17-AAG, directly binds to the ATP-binding pocket in the N-terminal domain of Hsp90 and, hence, blocks the binding of nucleotides to Hsp90. Once Hsp90 activity is inhibited, the Hsp90-dependent protein dissociates from the multi-chaperone complexes and is targeted for degradation by the ubiquitin-proteasome system [10,11]. 17-AAG-induced apoptosis has been previously reported to be associated with c-FLIP_S_ down-regulation [12]. However, the relationship between Hsp90 and c-FLIP_L_ has not been well elucidated.

C-terminus of Hsp70-interacting protein (CHIP) has the E3 ligase activity, and it binds to Hsp/Hsc70 and Hsp90 complex by means of its TPR (an amino-terminal tetratricopeptide) domain [13]. It has been reported that CHIP degrades Hsp90 client proteins, such as the glucocorticoid receptor, the cystic fibrosis transmembrane-conductance regulator and ErbB2 [13-15]. In this study, we show that CHIP is involved in c-FLIP_L_ degradation induced by Hsp90 inhibition. Since both Hsp90 and c-FLIP_L_ play a critical role in oncogenesis and chemoresistance, inhibition of Hsp90 by 17-AAG in combination with therapeutic agents targeting c-FLIP may be an efficacious strategy in patients with tumors that express high levels of c-FLIP_L_ and other Hsp90 client proteins.

## Results

### Hsp90 inhibitor 17-AAG and GA induces c-FLIP_L_ down-regulation via ubiquitin-proteasome pathway in NSCLC cells

**Figure 1:**
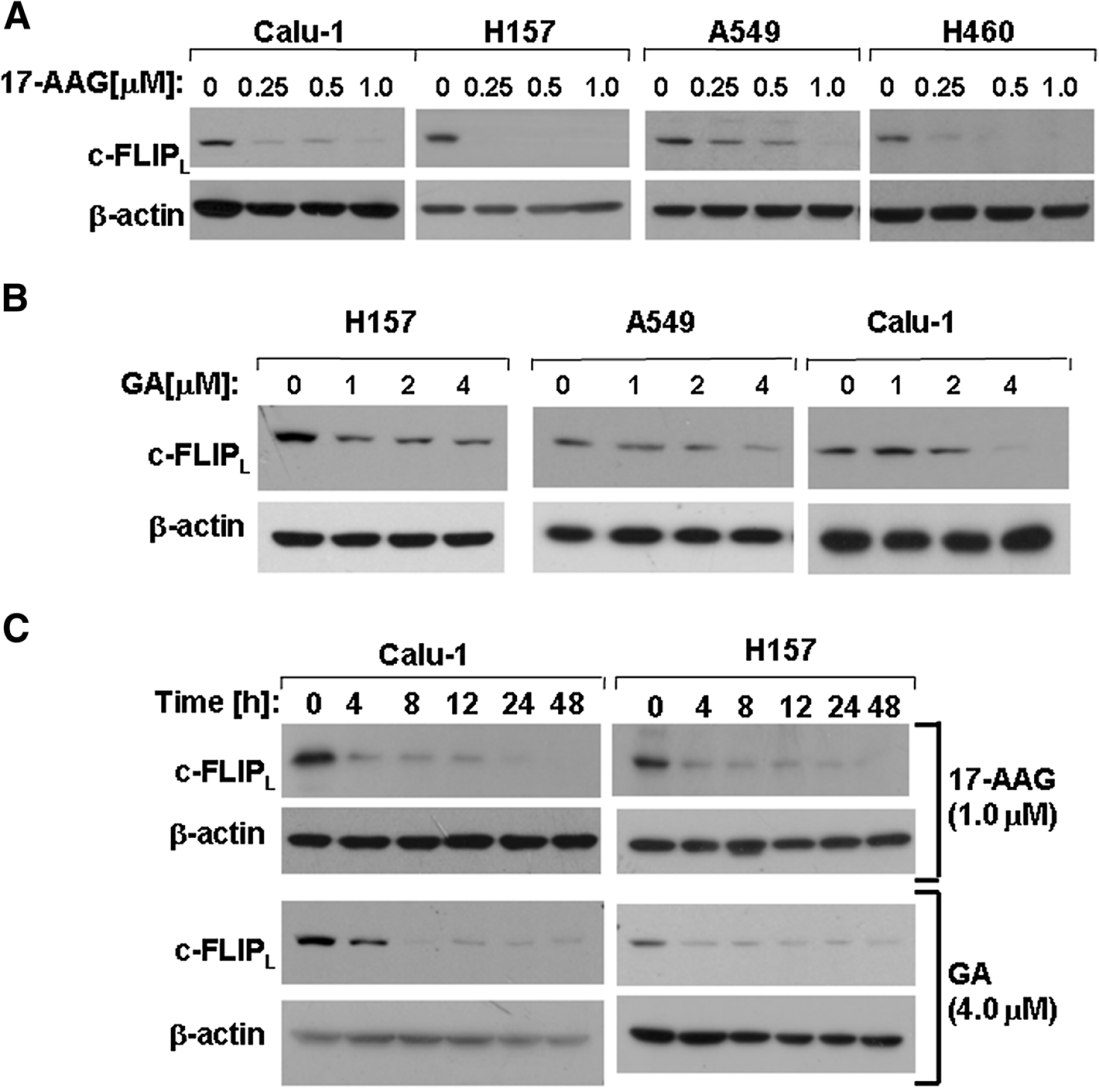
**17**-**AAG and GA induces c**-**FLIP**_**L **_**down**-**regulation in NSCLC cells.** (**A**) and (**B**), Calu-1, H157, A549 and H460 cell lines were treated with either 17-AAG or GA at the indicated concentration for 48 h. (**C**), Calu-1 and H157 cells were treated with 1.0 μM 17-AAG or 4.0 μM GA for the indicated time. The cells were subjected to preparation of whole-cell protein lysates and c-FLIP_L_ proteins were detected by Western blot analysis.

17-AAG-induced apoptosis has been previously reported to be associated with c-FLIP_S_ down-regulation [12]. However the relationship between 17-AAG/GA and c-FLIP_L_ has not been well elucidated. In this study we examined the effect of c-FLIP_L_ expression induced by 17-AAG or GA in lung cancer cell lines including Calu-1, A549, H460 and H157. After treatment with 17-AAG and GA at the indicated concentrations for 48 h, c-FLIP_L_ levels were reduced (Figure 1A and B). We also performed time course experiments to monitor the expression of c-FLIP_L_. Calu-1 and H157 cells were treated with 17-AAG (1.0 μM) or GA(4.0 μM) for the indicated time. We found that the c-FLIP_L_ expression decreased in a time-dependent manner. In Calu-1 and H157 cells, the time of c-FLIP_L_ down-regulation occurred as early as 4 h and was sustained for at least 48 h following 17-AAG or GA exposure (Figure 1C).

**Figure 2:**
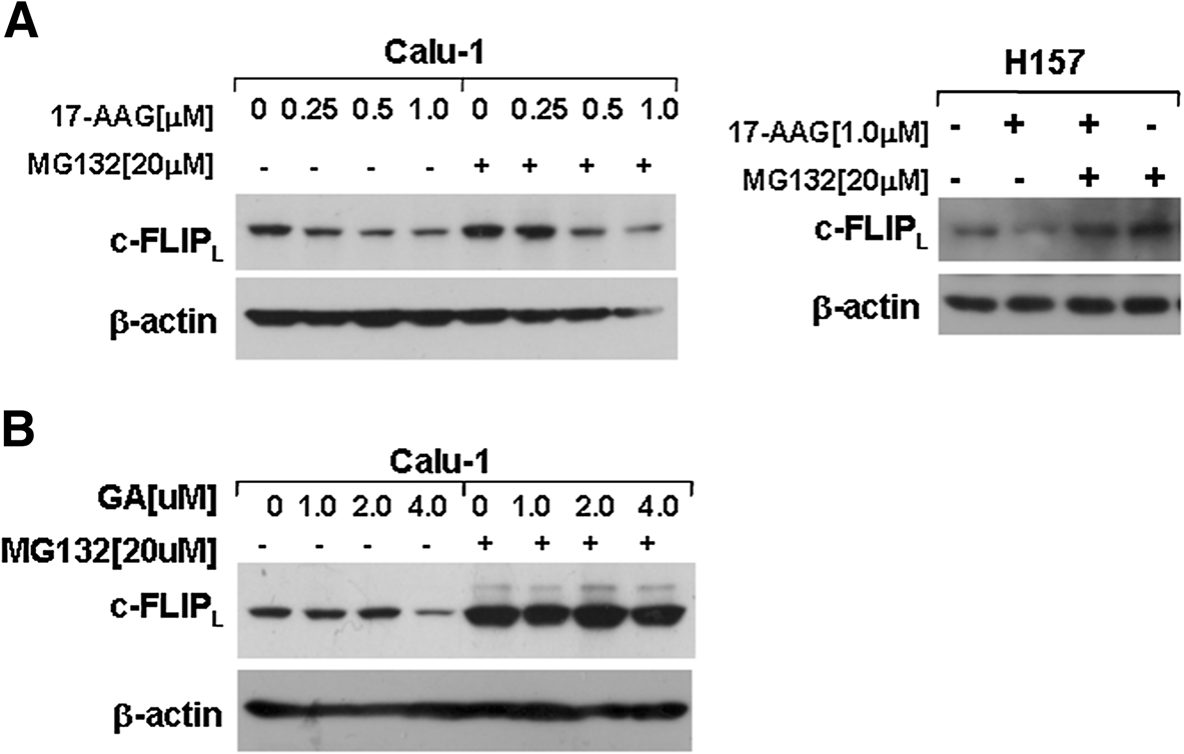
**17**-**AAG and GA induce c**-**FLIP**_**L **_**down**-**regulation via proteasome pathway.** Calu-1 and H157 cells were pretreated with 20 μM MG132 for 30 min and co-treated with the indicated concentrations of 17-AAG (**A**) or GA (**B**) for 4 h, and then the cells were subjected to preparation of whole-cell protein lysates and c-FLIP_L_ proteins were detected by Western blot analysis.

It has been reported that c-FLIP_L_ degradation is correlated with ubiquitin [7]. To determine whether 17-AAG induces proteasome-mediated c-FLIP_L_ degradation, we tested the effects of 17-AAG and GA on c-FLIP_L_ expression in the absence or presence of the proteasome inhibitor MG132 in Calu-1 and H157 cells. As shown in Figure 2A and B, MG132 at the concentration of 20 μM abrogated the ability of 17-AAG and GA to reduce c-FLIP_L_, suggesting that 17-AAG- and GA-induced c-FLIP_L_ down-regulation is dependent on proteasome-mediated degradation pathway.

### Inhibition of Hsp90 expression induces c-FLIP_L_ down-regulation

**Figure 3:**
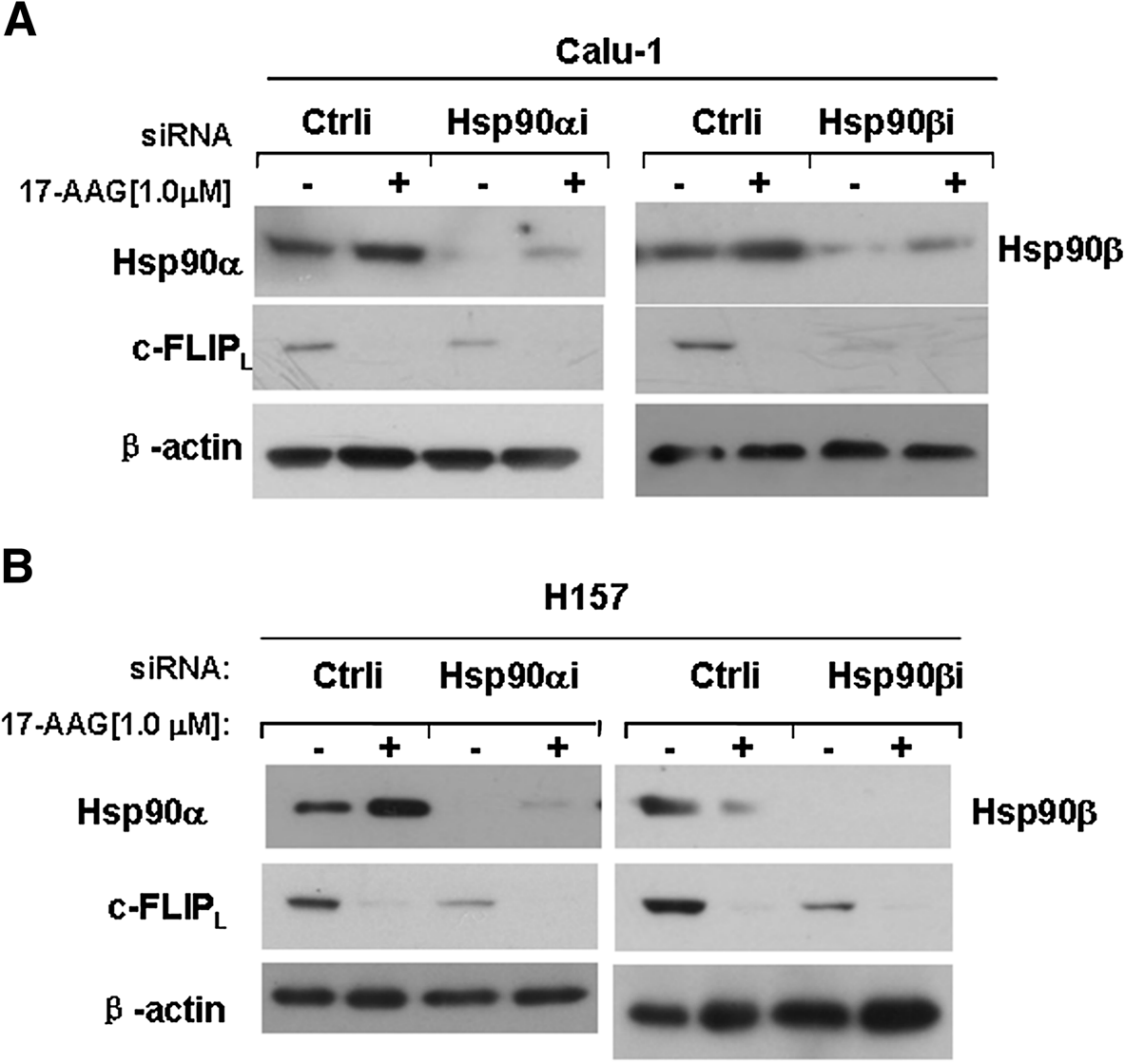
**Inhibition of Hsp90 expression facilitates c**-**FLIP**_**L **_**down**-**regulation.** (**A**) and (**B**), Calu-1 and H157 cells were transfected with siRNAs of control, Hsp90α and Hap90β, respectively. Twenty four hours later, cells were treated with 1.0 μM 17-AAG for 48 h. The cells were harvested for Western blot analysis to detect Hsp90α/β and c-FLIP_L_ levels.

It has been documented that 17-AAG and GA induced degradation of Hsp90 client proteins [9]. Since we detected that c-FLIP_L_ down-regulation was induced by 17-AAG and GA, we wondered whether c-FLIP_L_ was a client protein of Hsp90. We knocked down Hsp90α/β with siRNA and then detected the c-FLIP_L_ and Hsp90α/β protein levels by Western blot. Hsp90α/β protein levels were reduced after knockdown, and the c-FLIP_L_ level was also decreased compared to the control (Figure 3A and B), suggesting Hsp90 controls the stability of c-FLIP_L_ protein.

### CHIP modulates the c-FLIP_L_ degradation induced by 17-AAG

**Figure 4:**
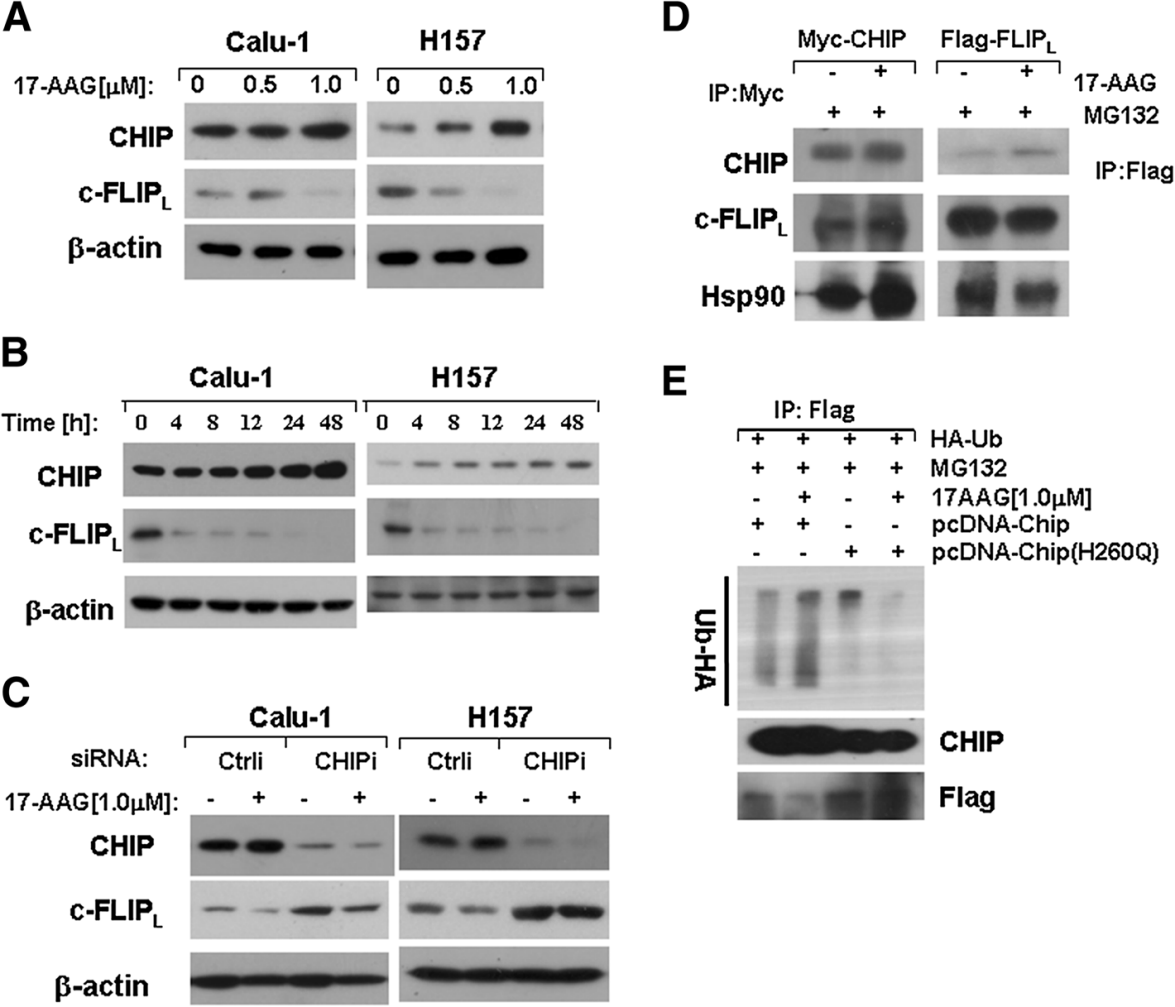
**CHIP is involved in c**-**FLIP**_**L **_**degradation.** (**A**), Calu-1 and H157 cells were treated with the indicated concentrations of 17-AAG for 48 h. (**B**), Calu-1 and H157 cells were treated with 1.0 μM 17-AAG for the indicated time. (**C**), Calu-1 and H157 cells were transfected with control siRNA (Crtli) and CHIP siRNA (CHIPi). 24 h after transfection, cells were reseeded and treated with 1.0 μM 17-AAG for 48 h. (**D**), Calu-1 cells were transfected with pcDNA-Myc-CHIP plasmid, and the cells were treated with 20 μM MG132 and 1.0 μM 17-AAG for 4 h. For immunoprecipitation, cell lysate was incubated with Myc antibody for 1 h and then incubated with protein A/G agarose(1:1 mix) at 4°C overnight(Left panel). Calu-1 cells were transfected with Lenti-Flag-c-FLIP_L_, and the cells were treated with 1.0 μM 17-AAG and 20 μM MG132 for 4 h. For immunoprecipitation, cell lysate was incubated with Anti-FLAG M2 beads at 4°C overnight (Right panel). The precipitated proteins were analyzed by Western Blot assay. (**E**), H157/c-FLIP_L_ cells were transfected with plasmids Ub-HA and pcDNA-Myc-CHIP or pcDNA-Myc-CHIP(H260Q mutant), and then the cells were treated with 1.0 μM 17-AAG and 20 μM MG132 for 8 h. The cell lysates were subjected to immunoprecipitation with Flag antibody and subsequently Western Blot analysis.

CHIP interacts with Hsp90 chaperone complexes and displays E3-ligase activity to degrade Hsp90 client proteins [13,15]. In order to detect the interaction of CHIP with c-FLIP_L_, we explored dose experiments in Calu-1 and H157 cells. We found that c-FLIP_L_ level was reduced while CHIP expression was slightly increased after treatment with 17-AAG (Figure 4A and B). Furthermore, when CHIP was knocked down, the degradation of c-FLIP_L_ was abrogated (Figure 4C) in Calu-1 and H157 cells, which indicated that c-FLIP_L_ degradation was modulated by CHIP.

### CHIP interacts with c-FLIP_L_ in vivo and promotes the ubiquitination of c-FLIP_L_

To further test if there is physically interaction between CHIP and c-FLIP_L_, immunoprecipitation experiments were performed. Calu-1 cells were transfected with plasmids carrying Myc-tagged CHIP or Flag-tagged c-FLIP_L_ and then treated with 1.0 μM 17-AAG for 4 h, the lysate supernatant was incubated with Myc antibody for 1 h and then with protein-G and protein-A (1:1 mix) beads overnight (left panel), or anti-FLAG M2 Affinity Gel beads overnight directly(right panel). Western Blot analysis showed that CHIP and c-FLIP_L_ proteins were both detected in the immunoprecipitation complexes, suggesting that both exogenous and endogenous CHIP interacts with c-FLIP_L_ physically in vivo. Hsp90 was also pulled down together with c-FLIP_L_ and CHIP, suggesting that CHIP interacts with c-FLIP_L_ in an Hsp90 chaperone complex (Figure 4D). Considering CHIP can be an E3 ligase, we wonder if it can regulate the ubiquitination level of c-FLIP_L_. H157-c-FLIPL cells were transfected with plasmids carrying Myc-tagged CHIP or Myc-tagged CHIP (H260Q Mutant) and then treated with 1.0 μM 17-AAG for 8 h. After immunoprecipitaion with Flag antibody, Flag-tagged c-FLIP_L_ was pulled down. Western Blot analysis showed that CHIP overexpression promoted the c-FLIP_L_ ubiquitination, while the mutant CHIP overexpression suppressed the c-FLIP_L_ ubiquitination after 17-AAG treatment (Figure 4E). Taken together, our data suggest that CHIP interacts with c-FLIP_L_ in vivo and promotes the ubiquitination of c-FLIP_L_.

### Ectopic overexpression of c-FLIP_L_ protects cancer cells from apoptosis

**Figure 5:**
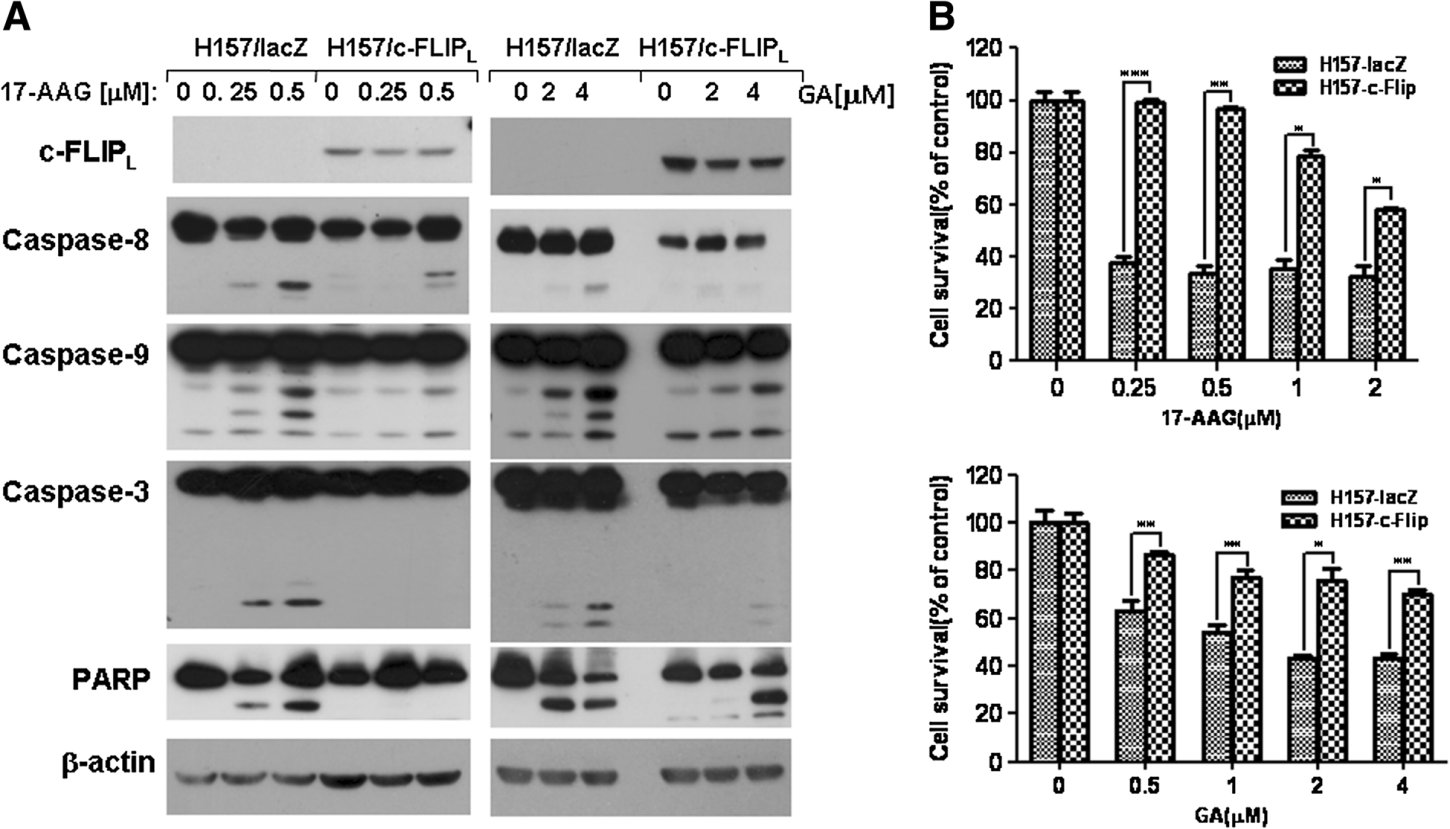
**Stable overexpression of exogenous c**-**FLIP**_**L **_**protects cells from 17**-**AAG**- **or GA**-**induced caspase activation and growth inhibition in human NSCLC cell lines.** (**A**), H157 cells stably expressing lacZ (H157-lacZ) and c-FLIP_L_ (H157-c-FLIP_L_) were treated with the indicated concentrations of 17-AAG or GA for 48 h and then the cells were subjected to Western blot analysis for detection of caspase activation and PARP cleavage. (**B**), H157-lacZ and H157-c-FLIP_L_ cells were cultured in the 96-well plates and then treated with the given concentrations of 17-AAG or GA for 48 h, then the cell number was estimated by SRB assay to determine the cell survival. Differences between groups are evaluated by Student’s *t*-test. *Columns*, mean of triplicate treatments; *bars*, ± SD. The statistical differences between the two treatments were analyzed by two-sided unpaired Student’s t tests (*P<0.05; **P<0.01; *** *P*<0.001).

To determine the effect of c-FLIP_L_ overexpression on 17-AAG- and GA-induced apoptosis, two cell clones H157-c-FLIP_L_ and H157-LacZ that overexpress c-FLIP_L_ and LacZ respectively were used to examine their effects on 17-AAG- and GA-induced caspase activation and apoptosis. By Western blotting, we detected high levels of ectopic c-FLIP_L_ in the H157-c-FLIP_L_ cell line. We found that 17-AAG or GA induced activation of caspase-8, caspase-9, caspase-3 and cleavage of PARP in H157-LacZ control cells, as demonstrated by increased levels of cleaved bands (Figure 5A). This was not seen in the corresponding H157-c-FLIP_L_ cells. We measured the survival of H157-LacZ to be 32.4% in cells treated with 2.0 μM 17-AAG for 48 h, whereas the survival of H157-c-FLIP_L_ cells were more than 58.1% (Figure 5B) by SRB assay. The similar result was observed for SRB assay in GA-treated cells. From the above results, we conclude that ectopic overexpression of c-FLIP_L_ protects cancer cells from apoptosis induced by Hsp90 inhibition.

### 17-AAG enhances celecoxib-induced c-FLIP_L_ down-regulation and apoptosis

**Figure 6:**
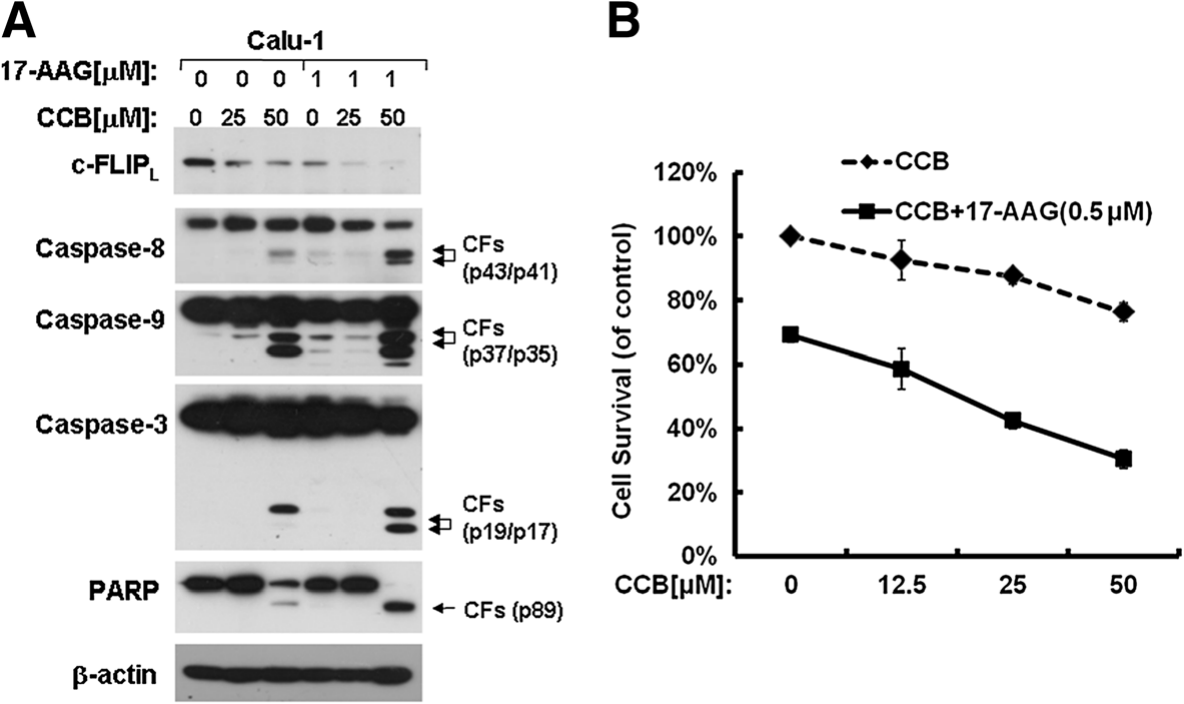
**17**-**AAG enhances celecoxib**-**induced c**-**FLIP**_**L **_**down**-**regulation and apoptosis.** (**A**), Calu-1 cells were treated with 17-AAG, celecoxib alone, and their respective combinations. After treatment for 48 h, the cells were subjected to detection of caspase activation and PARP by Western blot assay. (**B**), Calu-1 cells were seeded in 96-well plate and on the second day were treated with the given concentrations of CCB or combined concentrations of 17-AAG and CCB for 48 h, and then the cell number was estimated by SRB assay to determine the cell survival.

We have reported that celecoxib (CCB) down-regulates c-FLIP_L_ through ubiquitin-proteasome degradation [16]. To determine whether 17-AAG enhances celecoxib-induced c-FLIP_L_ down-regulation and apoptosis, we investigated the level of c-FLIP_L_ expression and caspase activation by combination treatment with CCB and 17-AAG in Calu-1 cells. We found that 17-AAG combination with CCB had synergistic effects on apoptosis induction by Western blotting and SRB assay (Figure 6A and B). c-FLIP_L_ expression decreased further when 17-AAG was combined with CCB compared with the monotherapy Calu-1 cells. In addition, the activation of caspase-8, caspase-9, caspase-3 and the cleavage of PARP were more pronounced. This data suggests that 17-AAG may enhance chemotherapeutic agent-induced c-FLIP_L_ down-regulation and apoptosis in lung cancer cells.

## Discussion

Hsp90 plays a key role in conformational maturation and stability of client proteins in transducing proliferative and anti-apoptotic signals [10]. In the present study we found that Hsp90 inhibitor 17-AAG and GA or Hsp90 knockdown induced c-FLIP_L_ down-regulation which subsequently resulted in cancer cell apoptosis. Although DR5 is an important death receptor in the extrinsic apoptotic pathway, in the above experiments, we failed to detect DR5 up-regulation (data no shown). This indicates that c-FLIP_L_ may primarily contribute to Hsp90 inhibition-induced apoptosis in lung cancer cells.

Currently, more than one hundred client proteins of Hsp90 have been identified, and they are degraded when Hsp90 multi-chaperone complex activity is disrupted by the inhibitors. When we inhibited Hsp90 expression using siRNA technique and examined the c-FLIP_L_ level, we found c-FLIP_L_ was reduced in the cells whose Hsp90 was knocked down, suggesting that c-FLIP_L_ is a client protein of Hsp90. It is reported when Hsp90 is inhibited, it recruits E3 ligase (e.g. CHIP) to degrade the client proteins [13-15]. When we knocked down CHIP, c-FLIP_L_ degradation was inhibited after treatment with 17-AAG, which indicated that CHIP modulated c-FLIP_L_ degradation in the NSCLC cell lines. Next, we performed immunoprecipitation experiments in Calu-1 cell lines. We found that CHIP was co-precipitated with c-FLIP_L_ in the precipitated complex. We also found that Hsp90 was precipitated at the same time, hinting that c-FLIP_L_ is the client protein of Hsp90 and CHIP modulates c-FLIP_L_ degradation through the Hsp90 chaperone complex. Itch is reported as an E3 ligase of c-FLIP_L_ in the mouse cells upon exposure to TNFα [7]. However, it has also been reported that some agents induced c-FLIP_L_ degradation independently of Itch [17-19]. Additionally, we found that CHIP promotes the ubiquitination of c-FLIP, indicating CHIP regulates c-FLIP_L_ degradation through uibiquitination when the cells were treated with 17-AAG.

It has been investigated that the clinically used anti-inflammatory drug celecoxib induces c-FLIP_L_ down-regulation and facilitates cancer cell apoptosis alone or in combination with other agents [16]. We found that 17-AAG and celecoxib had synergistic effects in promoting c-FLIP_L_ down-regulation and cellular apoptosis. In addition, overexpression of c-FLIP_L_ could rescue cancer cells from apoptosis. These data suggest that c-FLIP_L_ down-regulation plays important role in Hsp90 inhibition-induced apoptosis in NSCLC cells.

## Conclusions

Our data reveals that c-FLIP_L_ is down-regulated and apoptosis is induced effectively by Hsp90 inhibition, either by 17AAG and GA or siRNA technique, and c-FLIP_L_ is degraded via proteasome-mediated pathway. CHIP modulates c-FLIP degradation induced by Hsp90 inhibition in human NSCLC cell lines. Targeting both c-FLIP_L_ and Hsp90 may represent a promising therapeutic strategy for the patients whose tumors overexpress c-FLIP_L_ and Hsp90 client proteins.

## Materials and methods

### Reagents and antibodies

17-AAG and GA were purchased from LC Laboratories (Pompano Beach). Celecoxib, MG132 and Anti-FLAG M2 Affinity Gel were purchased from Sigma (St. Louis, MO). Protein A and Protein G Agarose were purchased from Roche (Mannheim, Germany).

Mouse anti-caspase-3 and anti-caspase-8 monoclonal antibodies, rabbit anti-caspase-9 and anti-poly (ADP-ribose) polymerase (PARP) antibodies were purchased from Cell Signaling Technology (Danvers, MA). Mouse anti-FLIP_L_ monoclonal antibody was purchased from Alexis Biochemicals (San Diego, CA). Mouse anti-Hsp90 antibodies were purchased from Abcam (Cambridge, UK). Mouse anti-Myc and anti-β-actin antibodies, rabbit anti-Flag and anti-CHIP antibodies were purchased from Sigma (St. Louis, MO).

### Cell lines, cell culture and transfection

The human non-small cell lung cancer cell lines used in this study were obtained from the American Type Culture Collection (Manassas, VA) and were grown in RPMI 1640 supplemented with 5% fetal bovine serum at 37°C in a humidified atmosphere of 5% CO_2_ and 95% air. Cells were transfected with different plasmids by using the FuGene reagent from Roche (Indianapolis, IN), according to the manufacturer’s instructions.

### Cell survival assay

Cells were seeded in 96-well plate with an appropriate amount in 100 μL culture medium per well. On the second day, compounds or drugs were mixed in and the total volume of per well was 200 μL. Cells were treated for defined time and then cell viability was measured by sulforhodamine B (SRB) assay as described previously [20].

### Western blot analysis

The whole-cell protein lysates preparation and Western blot analysis were described in reference [21].

### Plasmids and establishment of stable cell lines that overexpress c-FLIP_L_

Plasmids of Myc-CHIP and Myc-CHIP (H260Q) were kindly provided by Dr. Hamid Band (University of Nebraska Medical Center, USA). Lenti-Flag-c-FLIP_L_ was constructed before [22]. The cell clones that can overexpress c-FLIP_L_ and LacZ were constructed as previously described [22].

### Knockdown of Hsp90, CHIP expression with siRNA

siRNAs were synthesized by GenePharma (Shanghai, China). Control siRNA and Hsp90α/β siRNA target sequences were described before [23]. The target sequence of CHIP siRNA was 5^′^-AGGCCAAGCACGACAAGTA-3^′^. Transfection of siRNA was conducted as previously described [22].

### Immunoprecipitation

To precipitate Flag-tagged FLIP_L_ or Myc-tagged CHIP proteins, cells were lysed with lysis buffer and Anti-FLAG M2 Affinity Gel beads or Myc antibody with Protein A/G Agarose beads (1:1 mix) were used to pull down Flag-tagged FLIP_L_ or Myc-tagged CHIP proteins according to the manufacture’s instructions.

## Competing interests

The authors declare that they have no competing interests.

## Authors’ contributions

XGL and LS conducted the project design. QLW, WDS, XXH, TLL and LS conducted the experiments and data analysis. QL W and XG L drafted the manuscript. XG L managed the funding acquisition, supervised the project and was involved in interpretation of data and revision of the manuscript. All authors have contributed and approved the final manuscript.
